# Bone marrow-derived mesenchymal stem cells transplanted into a vascularized biodegradable tube containing decellularized allogenic nerve basal laminae promoted peripheral nerve regeneration; can it be an alternative of autologous nerve graft?

**DOI:** 10.1371/journal.pone.0254968

**Published:** 2021-08-31

**Authors:** Hiroki Tanaka, Ryosuke Kakinoki, Yukitoshi Kaizawa, Hirofumi Yurie, Ryosuke Ikeguchi, Masao Akagi

**Affiliations:** 1 Department of Orthopedic Surgery, Kindai University Hospital, Osakasayama, Osaka, Japan; 2 Department of Orthopedic Surgery, Graduate School of Medicine, Kyoto University, Sakyo-ku, Kyoto, Japan; University of Minnesota Medical School, UNITED STATES

## Abstract

Previously, we showed silicone nerve conduits containing a vascular bundle and decellularized allogenic basal laminae (DABLs) seeded with bone marrow-derived mesenchymal stem cells (BMSCs) demonstrated successful nerve regeneration. Nerve conduits should be flexible and biodegradable for clinical use. In the current study, we used nerve conduits made of polyglycoric acid (PGA) fiber mesh, which is flexible, biodegradable and capillary-permeable. DABLs were created using chemical surfactants to remove almost all cell debris. In part 1, capillary infiltration capability of the PGA tube was examined. Capillary infiltration into regenerated neural tissue was compared between the PGA tube with blood vessels attached extratubularly (extratubularly vascularized tube) and that containing blood vessels intratubularly (intratubularly vascularized tube). No significant difference was found in capillary formation or nerve regeneration between these two tubes. In part 2, a 20 mm gap created in a rat sciatic nerve model was bridged using the extratubularly vascularized PGA tube containing the DABLs with implantation of isogenic cultured BMSCs (TubeC+ group), that containing the DABLs without implantation of the BMSCs (TubeC- group), and 20 mm-long fresh autologous nerve graft (Auto group). Nerve regeneration in these three groups was assessed electrophysiologically and histomorphometrically. At 24 weeks, there was no significant difference in any electrophysiological parameters between TubeC+ and Auto groups, although all histological parameters in Auto group were significantly greater than those in TubeC+ and TubeC- groups, and TubeC+ group demonstrated significant better nerve regeneration than TubeC- group. The transplanted DABLs showed no signs of immunological rejection and some transplanted BMSCs were differentiated into cells with Schwann cell-like phenotype, which might have promoted nerve regeneration within the conduit. This study indicated that the TubeC+ nerve conduit may become an alternative to nerve autograft.

## 1. Introduction

Autologous nerve grafting is a gold standard for repair of peripheral nerve deficits. However, that is associated with the limit of nerve source and followed by neurological deficits in the area innervated by the donor nerves. Various artificial nerve conduits or allogenic nerves have been invented as substitutes for autologous nerve grafts and some of them are commercially available. However, their clinical use is still limited compared with autologous nerve grafts, especially in reconstruction of motor nerves or/and injuries with a long nerve tissue deficiency [1]. Kaizawa et al. demonstrated excellent nerve regeneration through a silicone tube containing blood vessels and decellularized allogenic nerve basal laminae (DABLs) seeded with isogenic bone marrow-derived mesenchymal stem cells (BMSCs) in the tubular lumen using a rat sciatic nerve model with a 20 mm interstump gap [2]. They mentioned that the DABLs showed no signs of immunological rejection and functioned as a scaffold for nerve fiber extension, Schwann cell migration and transplanted BMSC retention. Because the DABLs they used were made by the thermal technique [3–5], they contained a lot of cell debris inside, which leaves the possibility of pathologic organism transmission through the DABL transplantation. In addition, the nerve conduit was a silicone tube, which is solid, not biodegradable and capillary-permeable. From the clinical point of view, the wall of nerve conduits should be flexible to tolerate the joint motion, biodegradable and capable of capillary infiltration.

In the present study, we used the outer tubular part of Nerbridge (Toyobo Co., Ltd.; Osaka, Japan) as a nerve conduit, which was a soft and biodegradable tube made of polyglycolic acid (PGA) fiber mesh. The DABLs were created using chemical surfactants [6] to minimize the cell debris remaining within the DABLs to reduce the chance of pathologic organism transmission. In the first part of this study, we investigated the capillary infiltration capability into nerves regenerated through the PGA tube. In the second part, we bridge a 20 mm rat sciatic nerve deficit using an extratubularly vascularized PGA tube containing the chemically created DABLs seeded with BMSCs, and compared nerve regeneration through the tube with that through a 20 mm-long fresh nerve autograft. We hypothesized that the nerve regeneration within the vascularized PGA tube with DABL and BMSC transplantation would be equal to that in the fresh nerve graft because the DABLs would not only act as a scaffold of axon extension but also retain BMSCs, which would survive within the tube with the aid of blood supply through the PGA fiber wall and differentiate into Schwann cell-like cells within the tubular lumen filled with neurochemical factors secreted from the nerve stumps.

## 2. Materials and methods

This study was approved by the President of Kindai University after a review by Institutional Animal Care and Use Committee (approval numbers KAME-26-054 and KDMS-29-018) and carried out according to the guidelines of the Animal Research Committee of Kindai University Hospital.

### 2.1. Tubes

Nerbridge (Toyobo Co., Ltd.; Osaka, Japan) is an FDA-approved nerve inducing conduit and consists of an outer cylindrical part composed of braided PGA fibers coated with 1% w/v collagen hydrochloride solution and an inner core part made of collagen sponge. Nerbridge is thus flexible and biodegradable and dissolved about 3 months in vivo (Brochure Nerbridge Version 1; http://www.toyobo-global.com/news/pdf,2018/08/press20180820atch.pdf.). This tube has already been clinically used as a nerve autograft alternative and for treatment of neurologic pain [7–9]. In the current study, only the outer cylindrical part was used as a nerve conduit. The outer cylindrical part has a semipermeable feature that passes particles less than 600kDa (Brochure Nerbridge Version 1; http://www.toyobo-global.com/news/pdf,2018/08/press20180820atch.pdf.). We used the outer tubular part of Nerbridge with 2 mm or 3 mm inner diameters in this study. This material was kindly supplied by Toyobo Co., Ltd. (Osaka, Japan).

### 2.2. Animals

In this study, inbred female Lewis (LEW, RT-1^l^) rats (9–11 weeks old, weighing 180-220g) and inbred female Dark Agouti (DA, RT-1^a^) rats (9–11 weeks old, weighing 160-180g) were used. As donors of BMSCs, inbred female Lewis (LEW, RT-1^l^) rats (7 weeks old, weighing 170g) were used. These inbred Lewis and DA rats were purchased from Nippon SLC (Hamamatsu, Japan). Inbred Lewis rats (9–11 weeks old, weighing 160-180g) that expressed green fluorescent protein (GFP) [LEW-Tg(CAG-EGFP)1Ys] were purchased from National Bio-Resouce Project (Tokyo, Japan). All animals were acclimatized before surgical procedures, housed in flat-bottomed cages postoperatively, and allowed standard rat chow and water ad libitum. After the experiments, we performed euthanasia on all rats with intraperitoneal injection of overdose of sodium pentobarbital.

#### 2.2.1. Capillary permeability and nerve regeneration capability of the PGA tube

*2*.*2*.*1*.*1*. *Animals*. Thirty-three female Lewis rats were used in this study. Nine rats were used for immunohistochemical study and the remaining 24 rats were used for electrophysilogical and histomorphometric studies.

**Fig 1:**
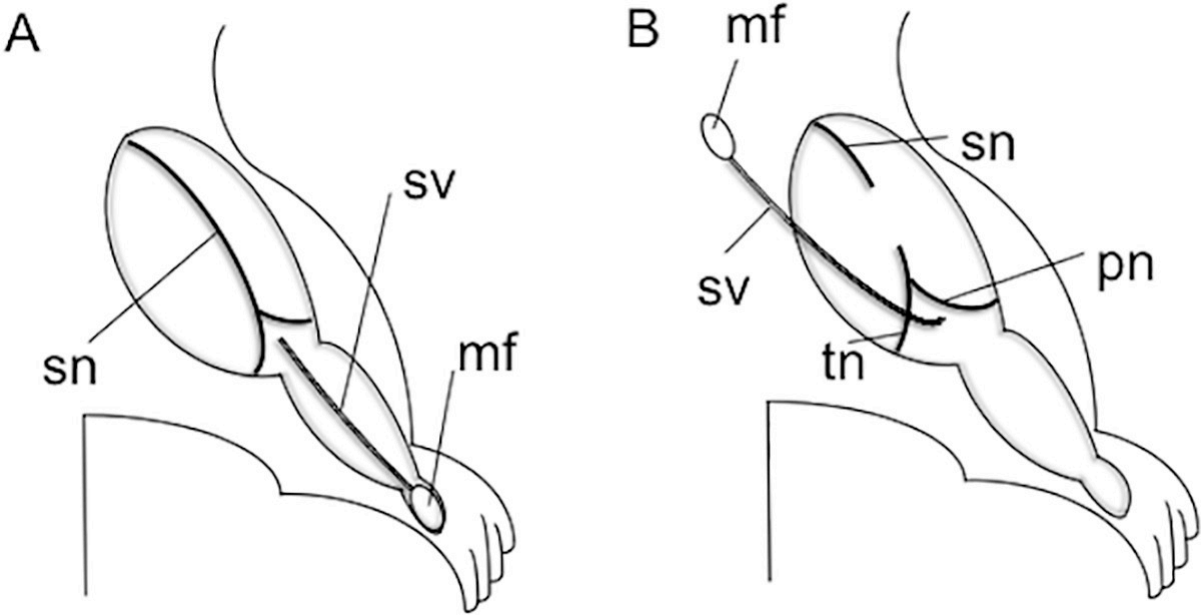
Surgical Procedures for elevating a flap nourished by the sural vessels. A; a 2mm X 5mm elliptical shaped flap (**mf**) vascularized by the sural vessels (**sv**) was elevated in the posterior surface of the ankle. B: The sciatic nerve (**sn**) was transected at the mid-thigh level. The flap (**mf**) was turned proximally with the origin of the sural vessels from the popliteal vessels as a pivot. **sv**; sural vessels. **mf**; monitor myocutaneous flap. **sn**; sciatic nerve. **pn**; peroneal nerve. **tn**; tibial nerve.

*2*.*2*.*1*.*2*. *Experimental models*. The rats were anesthetized with intepenetrial injection of sodium pentobarbital (40 mg/Kg) assisted by inhalation of isoflurane in oxygen for maintenance. A myocutaneous flap with a sural vascular pedicle was harvested as Kakinoki et al. reported previously [10]. In brief, a 3 cm skin incision was placed from the popliteal fossa to the ankle. The sural nerve was transected both just distal to its trifurcation from the sciatic nerve at the popliteal fossa and at the ankle, separated from its accompanying vessels (sural artery and vein), and removed completely. A 2 mm × 5 mm myocutaneous flap vascularized by the sural vessels was elevated from the posterior surface of the hind limb (Fig 1A). The incision was then extended to the hip joint, with the sciatic nerve exposed from the sciatic notch to the popliteal fossa, using a gluteal muscle-splitting approach. The sciatic nerve was transected in the middle of the thigh (Fig 1B).

The PGA tubes with 8 mm in length and 2 mm in an inner diameter were used in the following E-tube, I-tube and N-tube groups.

**Fig 2:**
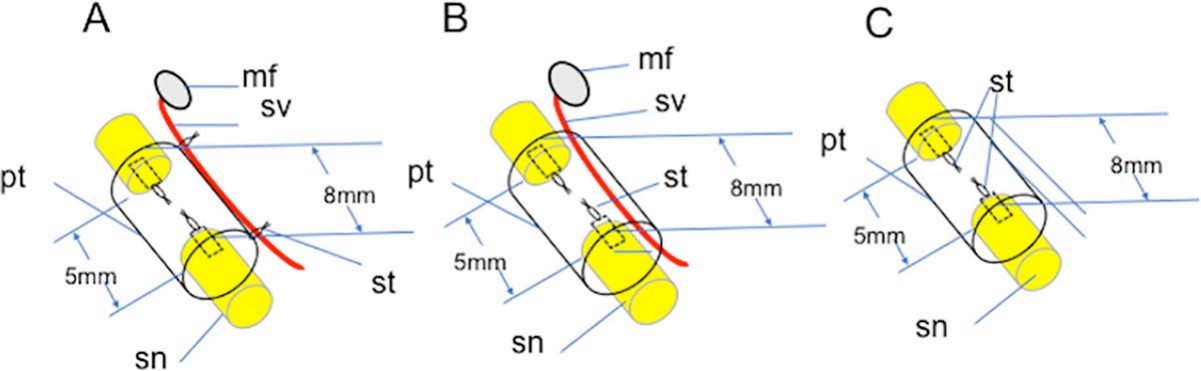
Surgical procedures of E-tube, I-tube and N-tube groups. **A:** E-tube group. The sural vascular bundle (**sv**) harvested from the posterior surface of the ankle was attached on the outer surface of the PGA tube (**pt**) using 2 stiches of a 10–0 nylon suture (**st**). The transected sciatic nerve stumps (**sn**) were joined to either end of the tube leaving a 5 mm gap using a 10–0 nylon suture (**st**). The tube (**pt**) and the sural vascular pedicle (**sv**) were wrapped with a thin water-nonpermeable plastic sheet with 20 mm × 20 mm. **B:** I-tube group. A 3–0 nylon suture placed in the distal end of the monitor flap (**mf**) was passed through the tube and pulled out proximally. The flap (**mf**) and vascular pedicle (**sv**) were thus passed through the tubular lumen. The transected sciatic nerve stumps (**sn**) were joined to either end of the tube leaving a 5 mm gap using a 10–0 nylon suture. The tube (**pt**) was wrapped with a water-nonpermeable plastic sheet with 20 mm × 20 mm. **C:** N-tube group. The transected sciatic nerve stumps were bridged by an 8 mm-long PGA tube (**pt**) leaving a 5 mm interstump gap. The tube was wrapped with a water-nonpermeable plastic sheet with 20 mm × 20 mm. No blood vessels were placed along the tube. The plastic sheets wrapping the tubes were not drawn in the schematic diagrams.

In E-tube group, the transected sciatic nerve stumps were sutured to either end of the PGA tube leaving a 5 mm interstump gap using a 10–0 monofilament nylon suture. The sural vascular pedicle was then placed along the tube. The connective tissue around the vascular pedicle was sutured to the outer surface of the tube using two 10–0 nylon suture stiches (Fig 2A).

In I-tube group, a 3–0 nylon suture was placed in the distal end of the myocutaneous flap harvested from the posterior surface of the ankle. The 3–0 suture was inserted in the tube and taken out from the opposite end of the tube. The flap and vascular pedicle were thus passed through the tubular lumen. The transected sciatic nerve stumps were joined to either end of the tube leaving a 5 mm gap using a 10–0 nylon suture (Fig 2B).

In N-tube group, the sciatic nerve stumps were sutured to either end of the 8mm-long tube leaving a 5 mm gap in the same way as E-tube group. However, no sural vessels were attached to the tube (Fig 2C).

Each tube was wrapped with a water-nonpermeable plastic sheet with 0.1 mm thickness and 20 mm × 20 mm width to shut down the capillary ingrowth from the surrounding tissue except the sciatic nerve stumps joined to the either end of the tube in all groups and the sural vessels in E-tube and I tube groups (Fig 2). For postoperative analgesia, intraoperitoneal fentanyl 0.02 mg/Kg was administered just after surgery.

*2*.*2*.*1*.*3*. *Evaluation of vascular supply to the regenerated nerve tissue within the tubes*. The revascularization of nerve tissue formed within a tube was investigated and compared among E-tube, I-tube and N-tube groups. Three rats each were used for this study. At 4 weeks, the revascularization of the midportion of each tube (which is theoretically the most ischemic part of the regenerated nerve within the tubes) of the three groups was evaluated immunohistochemically.

Three rats each of the three groups were sacrificed and the transplantation sites of the tubes were exposed. From the middle portion of each tube, a 3 mm segment was harvested. The segment was fixed in 4% paraformaldehyde (PFA) in 0.1M phosphate buffer (PB, pH 7.4) overnight at 4°C. They were then cryoprotected in 20% sucrose for 48 hours at 4°C. Next, the sample was embedded in optimal cutting temperature compound (OCT, Sakura Finetechnical, Torrance, CA), and three sequential 16 μm-thick transverse frozen sections were taken from each segment and placed onto poly-L-lysine-coated slides. Following 3 washes with PBS, antigen retrieval was performed in proteinase K (PK; Sigma-Aldrich) at room temperature for 10 minutes. For blocking procedure, donkey serum was added onto the slides and incubated at room temperature for 1 hour. Sections were incubated at 4°C for 48 hours with primary antibody, anti-rat endothelial cell cytoplasmic antigen (RECA-1) antibody (abcam, 1:40). Slides were then washed with PBS and incubated at room temperature for 1 hour with secondary antibodies, donkey anti-mouse IgG (H+L) whole antibody (1:200, CFTM543 fluorescent reagents; Biotium). After further PBS washing, cover slides were mounted onto the slides with bicarbonate buffered glycerol (pH 8.6), and the slides were viewed with a fluorescence microscope (KEYENCE BZ-9000; NKEYENCE, Osaka, Japan). Negative controls included omission of primary or secondary antibodies on parallel sections. The numbers of the RECA-1 positive cells were counted on a section among the three sequential sections of each segment, using captured images of five random high-powered fields per section.

*2*.*2*.*1*.*4*. *Nerve regeneration capability through the PGA tubes*. Twelve weeks after surgery, 8 rats each of the 3 groups were anesthetized. The right sciatic nerve was exposed and stimulated just distal to the piriformis muscle (S1) and at the popliteal fossa (S2) using a bipolar silver electrode. Two pairs of needle electrodes were inserted into the pedal adductor muscle to check for the presence of an evoked action potential. Motor nerve conduction velocity (MNCV) was calculated for both types of evoked action potential stimulated at S1 and S2. The amplitude (peak to peak) of the compound muscle action potentials (CMAPs) that were evoked in the pedal adductor muscles with the supramaximal electric stimulation at S1 point was measured. The same procedure was performed on the left intact hind limb. The results of CMAP amplitudes and MNCVs of the operated limb were expressed as a percentage of the values on the contralateral healthy limb [10].

After the electrophysiological study, the tubes and their adjacent nerve portions were removed, fixed in 1% glutaraldehyde and 1.44% paraformaldehyde, postfixed with 1% osmic acid, and embedded in epoxy resin. Transverse sections (1 μm thick) of the regenerating nerves were taken from the midpoint of each tube. The sections were stained with 0.5% (w/v) toluidine blue solution and examined by a light microscope (KEYENCE BZ-9000, KEYENCE, Osaka, Japan). The total number of regenerated myelinated axons was counted using Image J (NIH) (http://rsbweb.nih.gov/ij/), as reported in our previous studies [2, 11–13]. Briefly, the entire neural area (a) of each specimen was calculated on an image at a magnification of 40×. Six or 7 fields were chosen at random, so that the area analyzed would represent 20% of the entire neural area of each specimen. The number of myelinated axons and the neural area were calculated for each field at a magnification of 400×. The number of myelinated axons (b) and neural areas (c) from 6 or 7 fields were aggregated. The total number of myelinated axons in each specimen was estimated as b × a/c. Ultrathin sections of the same tissues stained with uranyl acetate and lead citrate were examined with a transmission electron microscope (TEM, HITACHI model H-7000) equipped with an image acquisition system at a magnification of 2000× for myelinated axon diameters (ADs) and myelinated nerve fiber diameters (NFDs). Photographs from 10 random fields of each ultrathin nerve section were analyzed by use of Image J [14–16]. Myelin thickness was calculated according to the following formula: (NFD-AD)/2. Mean myelinated axon number, axon diameter and myelin thickness were statistically compared among the groups.

#### 2.2.2. Comparison of nerve regeneration through extratubularly vascularized PGA tubes containing DABLs seeded with BMSCs (TubeC+ group), extratubularly vascularized PGA tubes containing DABLs without BMSC transplantation (TubeC- group) and fresh autologous nerve segments (Auto group) in rat sciatic nerve with a 20mm deficit

*2*.*2*.*2*.*1 Animals*. Seventy-five Lewis rats were used as recipients and two 7-week Lewis rats were used to source BMSCs. Fifty-four inbred female DA rats were used as donors of DABLs and fresh sciatic nerve segments. This rat strain combination represents a genetic difference of both major histocompatibility complex (MHC) and non-MHC [17]. Among them, four rats died during surgery or the postoperative follow-up period.

#### 2.2.3. Decellularized allogenic nerve basal laminae (DABLs)

*2*.*2*.*3*.*1*. *Preparation of DABLs*. Forty-eight DA rats were used to make 96 sciatic nerve DABL segments for the present study. Under aseptic condition, bilateral sciatic nerves of anesthetized DA rats were exposed. The entire sciatic nerves were excised and the epineurium of each nerve segment was removed. DABLs were prepared using the chemical surfactant extraction process, and all the subsequent steps were conducted based on the previously developed protocol [6]. In brief, nerve tissues were immersed in purified water at room temperature for 7 hours, which was followed by immersion in phosphate-buffered saline (PBS) containing 125mM SB-100 for 15 hours. After being rinsed in PBS for 15 minutes, the nerve tissue was put to PBS containing 0.6mM SB-16 and 0.14% TritonX-200 and immersed for 24 hours. The nerve tissue was rinsed with purified water for 5 minutes. This rinse was repeated 3 times. The tissue was transferred to PBS liquid containing 0.6mM SB-16 and immersed in it for 7 hours. After being immersed in purified water, it was immersed in PBS containing 0.6mM SB-16 and 0.14% TritonX-200 again for 15 hours. Finally, the tissue was rinsed for 15 minutes and dried at room temperature overnight. Then, the tissue was subjected to gamma ray exposure (20KGy/hour × 5 hours) and preserved in a refrigerator until use. The length of each DABL segment was set to 20 mm just before transplantation.

*2*.*2*.*3*.*2*. *Histological study of DABLs*: *reduction of the cellular component and preservation of laminin*. To assess the efficacy of reduction of cellular components and the preservation of laminin in the DABLs, electron microscopic examinations and immunohistochemical examination for laminin were performed on sections of the DABLs. As a control of the chemically created DABLs, we also made DABLs using a freeze and thaw technique, which was based on the previously developed protocol [3–5]. In brief, sciatic nerve segments harvested from DA rats were immersed in liquid nitrogen until thermal equilibrium was achieved, and then put into phosphate-buffered saline (PBS) for 2 minutes. This freezing and thawing cycle was repeated 3 times. The grafts were preserved in the freezer (-20°C) until use. The length of each DABLs was set to 20 mm just before the use.

Three each chemically and thermally created DABLs were used. From the middle portion of each DABL, three 3mm-long segments were harvested. For the transmission electron microscopic examination, one of the segments was fixed in 1% glutaraldehyde and 1.44% paraformaldehyde, postfixed with 1% osmic acid, and embedded in epoxy resin. Transverse sections (1 μm-thick) of the DABLs were taken from the midportion of the fragment. Ultrathin sections of the same tissues stained with uranyl acetate and lead citrate were created and examined with a transmission electron microscope (TEM, HITACHI model H-7000) at a magnification of 2000×. For the scanning electron microscopic examination, another 3mm-long DABL segment was used. The segment was fixed in 1% glutaraldehyde and 1.44% paraformaldehyde, postfixed with 1% osmic acid. A section was taken and dehydrated using series of ethanol liquids with different concentration and immersed in the mixture containing the same volume of 100% ethanol and t-butyl alcohol for 30 minutes. After that, the segment was immersed twice in t-butyl alcohol liquid for 15 minutes and then frozen in a freezing chamber. The segment was transferred to a vacuum lyophilization. After being coated with platinum-palladium, the section was examined using a scanning electron microscope (HITACHI SU3500, Tokyo, Japan) with 15KV acceleration electric volume at magnification 800×.

For the immunostaining, transverse 16 μm-thick frozen sections were obtained from the remaining DABL segment and prepared in the same way as described previously. The sections were placed onto poly-L-lysine-coated slides. Rabbit polyclonal anti-laminin antibody (1:10, Abcam, UK) was used as the primary antibody and donkey anti-rabbit IgG (H+L) whole antibody (1:200, CF^TM^488 fluorescent reagents, Biotium, Richmond, CA) was used as the secondary antibody. The slides were viewed with a fluorescence microscope (KEYENCE BZ-9000; NKEYENCE, Osaka, Japan).

To compare the removal of the cellular component between the chemically and thermally created DABLs, genomic DNA was extracted from 10 small pieces of each DABL using a DNA extraction kit (DNeasy Blood & Tissue Kit, Qiagen, Tokyo, Japan) and quantified spectrophotometrically. The amount of the extracted DNA was expressed as ng /mg dry DABL tissue weight.

#### 2.2.4. Preparation of isogenic bone marrow stromal cells (BMSCs)

BMSCs were obtained from 7 week-female inbred Lewis rats [18]. Briefly, the distal and proximal ends of the femoral and tibia bones were removed to expose bone marrow cavities. Mesenchymal cell growth medium (MEM-Alpha; Gibco; supplemented with 10% FBS and 1% penicillin-streptomycin) was injected through each cavity using a 21G needle. The resulting cell suspension was filtered through a 70 μm Falcon filter and centrifuged for 5 minutes at 1800 × g. The supernatant was aspirated and the cell bolus was resuspended in mesenchymal cell growth medium, plated in 75 cm^2^ tissue culture flasks and incubated in 5% CO_2_ at 37°C. The non-adherent cells were removed after 48 hours. Thereafter, the fresh mesenchymal cell growth medium was replaced every 3 days. When the cells reached 80–85% confluence, the cultures were treated with trypsin-EDTA solution (0.25% trypsin, 0.02% EDTA, Sigma, St. Louis, MO, US), then harvested and diluted to 1:4 per passage for further expansion. The cells at passage 5–7 were used for experiments [19].

#### 2.2.5. Surgery

**Fig 3:**
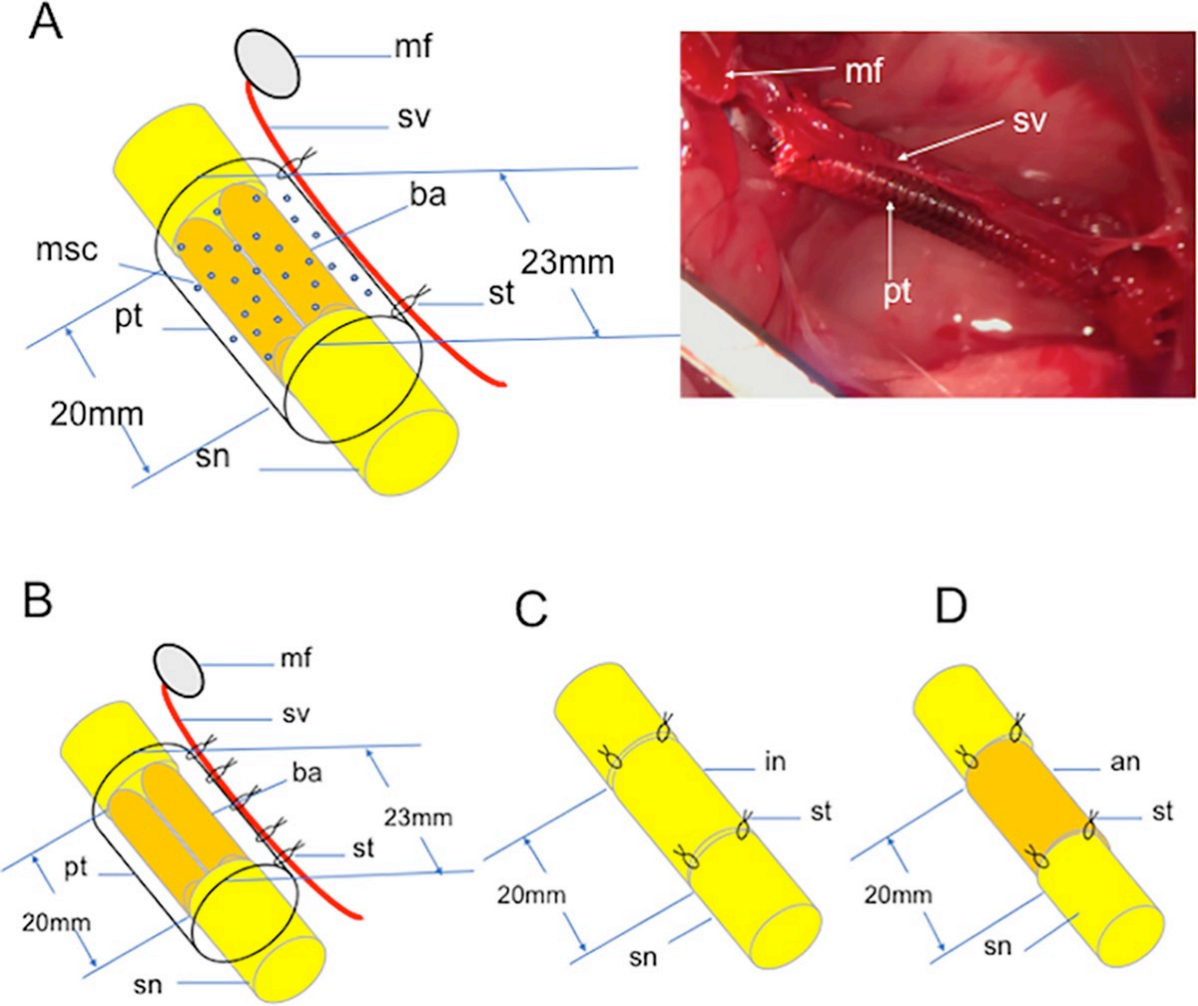
Schematic diagrams of TubeC+,Tube C-, Auto and Allo groups. **A**: A schematic diagram (left) and an intraoperative photo (right) of TubeC+ group. **B**: TubeC- group. **C**: Auto group. **D**: Allo group. **mf**; monitor flap, **sv**; sural vessels, **pt**; PGA fiber tube, **sn**; sciatic nerve, **st**; 10–0 nylon suture stich, **da**; DABLs, **sc**; BMSCs, **in**; reversed fresh isogenic nerve segment, **an**; reversed fresh allogenic nerve segment.

Lewis rats were divided into four surgical groups; TubeC+ group, TubeC- group, Auto group and Allo group (Fig 3A–3D). In TubeC+ and TubeC- groups, the 23 mm-long PGA tubes with a 3 mm inner diameter were used. A 15 mm segment of the sciatic nerve was removed at the mid-thigh level. Because the DABLs made from the sciatic nerve became almost half the thickness of the original sciatic nerve during the decellularized process, two 20 mm-long DABL segments prepared as mentioned above were inserted into the PGA mesh tube leaving a 1.5 mm-long tubular cuff on either end of the tube. Each transected sciatic nerve stump was joined to the inside of the tube for approximately 1.5mm from either end of the tube using two 10–0 monofilament nylon sutures so that the transected nerve stumps would contact the ends of the DABL segments transplanted in the tubular lumen. In TubeC+ group, the above mentioned BMSCs in 0.075 ml of α-MEM solution at a concentration of 3 × 10^6^ cells/ml [20] were injected into the DABL segments at three different points with a 5 mm interval each using a 27G needle [21] (Fig 3A). In TubeC- group, BMSCs were not transplanted into the tubular chamber. After that, in both groups, the pedicled myocutaneous flap was harvested from the lower leg and turned proximally with the origin of the sural vessels from the popliteal vessels as a pivot in the same way as described in Fig 1. The connective tissue around the sural vessels was sutured with the outer layer of the tube using five 10–0 nylon suture stiches with a 5 mm interval in the same way described in E-tube group in this study (Fig 3B). In both TubeC+ and TubeC- groups, the wound was washed with physiological saline and closed with 4–0 nylon sutures in layers. The myocutaneous flaps were sutured to the buttocks and used as monitor flaps to indicate whether the translocated sural vessels remained patent. No immunosuppressants were used in the perioperative period.

In Auto group, a right sciatic nerve was exposed from the sciatic notch to the popliteal fossa using a gluteal muscle-splitting approach. Then a 20 mm-long fresh nerve segment was removed. The nerve segment was reversed and sutured in situ (Fig 3C).

In Allo group, a 15 mm-segment of the right sciatic nerve was removed in a recipient Lewis rat. A 20 mm-long fresh sciatic nerve segment harvested from a DA rat was transplanted between the transected right sciatic nerve stumps of each Lewis rat using 10–0 nylon sutures (Fig 3D).

#### 2.2.6. Immunogenicity of DABLs

The immunogenicity of the DABLs transplanted in TubeC+ group was investigated in comparison with autologous sciatic nerve segments (Auto group; as a negative control) and unprocessed allogenic sciatic nerve segments (Allo group; as a positive control). Four Lewis rats each were assigned to each of the four groups. 16 DABL segments were taken from eight DA rats and four fresh sciatic nerve segments were taken from two DA rats. Right sciatic nerves of 16 Lewis rats were subjected to the recipients of the tubes and nerve segments. At 4 weeks, the immunogenicity of the graft was evaluated immunohistochemically. A 16 μm-thickness frozen section taken from the midportion of each DABL segment in TubeC+ and TubeC- groups and the nerve grafts in Auto and Allo groups underwent immunohistochemical staining for CD8a (surface marker on cytotoxic T cells) at 4 weeks [22]. The sections were incubated at 4°C for 24 hours with mouse monoclonal antibodies to CD8a (1:10, Abcam) as primary antibodies. Secondary antibodies employed were donkey anti-mouse IgG (H+L) whole antibody (1:200, CFTM488 fluorescent reagents; Biotium, Richmond, CA, USA). The numbers of the CD8a-positive cells were counted, using captured images of five random high-powered fields per section. The numbers of the CD8a-positive cells per section were expressed as per mm^2^ and the mean value of four rats in each group was compared among the four groups.

#### 2.2.7. Differentiation of BMSCs in TubeC+ group tubes

The differentiation of the BMSCs implanted into the tubes of TubeC+ group was histologically investigated 6 weeks after surgery.

*2*.*2*.*7*.*1*. *Preparation and transplantation of GFP positive BMSCs*. In this study, the BMSCs were obtained from two Lewis rats that expressed green fluorescent protein (GFP) [LEW-Tg(CAG-EGFP)1Ys], which allowed us to trace the cells after implantation.

Six Lewis rats were used. A 20mm-long sciatic nerve gap was created and bridged using a 23 mm-long extratubularly vascularized PGA tube with implantation of two 20 mm-long DABL segments described in Tube C- group (Fig 3B). Then GFP positive BMSCs in 0.075 ml of α-MEM solution at concentration of 3 x 10^6^ cells/ml were injected into the DABL segments in each tube using 27G needle at three different points with a 5 mm interval each in the same way as described in TubeC+ group (Fig 3A). No immunosuppressants were used in the perioperative period.

*2*.*2*.*7*.*2*. *Immunochemistry to identify cells demonstrating Schwann cell like phenotype with transplanted BMSC origin*. At 6 weeks, the midportion of each conduit in the rats was subjected to the following immunochemical staining. Following fixation in 4% PFA at 4°C overnight, several 16 μm-frozen sections were prepared as mentioned above and underwent immunohistochemistry for GFP and S-100 protein (S-100). Sections were incubated with primary antibodies, chicken monoclonal anti-GFP antibody (1:200, Abcam) and rabbit polyclonal anti-S-100 antibody (1:500, Dako, Carpinteria, CA, USA) at 4°C for 24 hours. Secondary antibodies used were donkey anti-chicken IgG (H+L) whole antibody [1:200, CFTM488 fluorescent reagents (for GFP); Biotium] and donkey anti-rabbit IgG (H+L) whole antibody [1:200, CFTM543 fluorescent reagents (for S-100); Biotium]. The sections were observed using a fluorescence microscope (KEYENCE BZ-9000; NKEYENCE, Osaka, Japan).

#### 2.2.8. Comparison of nerve regeneration among TubeC+, Tube C- and Auto groups

Electrophysiological and histomorphometric analyses were performed on eight rats each of TubeC+, TubeC- and Auto groups at 12 and 24 weeks. The mean values of each examined parameter in these groups were statistically compared at each time point.

*2*.*2*.*8*.*1*. *Electrophysiological evaluations*. Twelve and 24 weeks after surgery, the rats of the three groups were anesthetized and underwent electrophysiological study in the same way as described previously in this study. The results of CMAP amplitudes in the pedal adductor muscles and MNCVs were examined in the bilateral hind limbs. The value of the operated limb was expressed as a percentage of that of the contralateral healthy limb.

*2*.*2*.*8*.*2*. *Histological and morphometric evaluations*. After the electrophysiological study, the histological and morphometric evaluations (total myelinated axon number, axon diameter and myelin thickness) were performed on transverse sections taken 5 mm proximal to the distal sutures of the transplanted tubes or nerve segments in the same way as described previously in this study and statistically compared among three groups.

*2*.*2*.*8*.*3*. *Reinnervated muscle weight*. At 24 weeks, bilateral tibialis anterior muscles of the rats were dissected cleanly from the origin and insertion, then were removed and weighed immediately with an electron scale with 0.01 g precision. The results were expressed as a percentage of the values on the contralateral healthy side.

### 2.3. Statistical analysis

To determine the significant difference among groups, the data were analyzed with the Excel 2010 software package. For comparison of two sampled groups, Student’s t-test for the data of normal distribution was used. To multiple group comparisons, analysis of one-way ANOVA with Bonferroni correction was applied. Results were expressed as mean ± SD, and p values <0.05 were considered statistically significant.

## 4. Results

### 4.1. Capillary permeability and nerve regeneration capability of the PGA tube

#### 4.1.1. Revascularization of nerve tissue regenerated within tubes with a 5 mm interstump gap at 4 weeks

**Fig 4:**
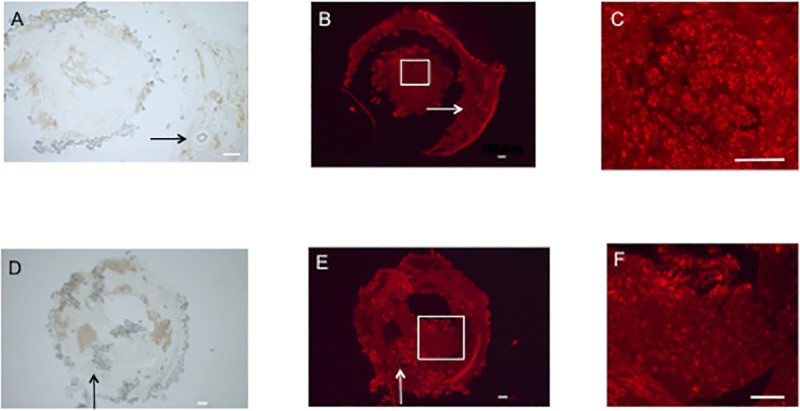
Revascularization of nerve tissue formed through E-tubes and I-tubes 4 weeks after the transplantation surgery. Immunohistochemistry for RECA-1 was performed on transverse sections of the midportion of the conduits in E-tube and I-tube groups. **A, B, C**; E-tube group. **D, E, F**; I-tube group. **A** and **D**; transverse sections with omission of the primary or secondary antibodies on parallel sections. **B** and **E**; immunostaining for RECA-1 with low magnification. The square shaped areas in **B** and **E** were magnified in **C** and **F**, respectively. **C** and **F**; immunostaining for RECA-1 with high magnification. Arrows indicate the transplanted vascular bundles. Bar = 100μm.

Three sections (one section per animal) were observed in each group. In E-Tube group, many RECA-1positive cells were densely and diffusively observed in the tube material around the attached site of the transplanted vascular bundle as well as in the intratubular regenerated nerve tissue (Fig 4A–4C). In I-tube group, RECA-1 positive cells were located densely around the vascular bundle and diffusively in the entire regenerated nerve tissue (Fig 4D–4F). The mean number ± standard deviations of RECA-1 positive cells were 175±37 and 162±29 in E-tube and I-tube groups, respectively at 4 weeks after surgery. In N-tube group, RECA-1 staining was not performed because no neural tissue was found between the nerve stumps in any rat.

#### 4.1.2. Electrophysiological evaluations of nerve regeneration through the three tubes

All rats successfully evoked compound muscle action potentials in the pedal adductor muscles in the operated limbs in E-tube and I-tube groups, while no action potential was evoked in any rat in N-tube groups at 12 weeks. Because no rats evoked action potentials in the pedal adductor muscles in N-tube group, statistical comparison was performed between E-tube and I-tube groups. There was no significant difference in the mean MNCVs or mean CMAP amplitudes between I-tube and E-tube groups (Table 1).

**Table 1:** Electrophysiological and histomorphometric studies of I-Tube and E-Tube groups at 12 weeks.

*Parameters*	*I-Tube*	*E-Tube*	*p value*
*MNCV*	0.34±0.11	0.48±0.18	0.56
*CMAP*	0.12±0.04	0.11±0.03	0.08
*Axon Number*	6162±1867	5712±930	0.53
*Axon Diameter (μm)*	2.19±0.28	2.17±0.42	0.9
*Myelin Thickness (μm)*	0.74±0.15	0.69±0.09	0.35

Note: Each value expresses the mean value ± standard deviation. The MNCVs and　CMAP amplitudes were expressed as the ratios of those of the contralateral healthy limbs.

#### 4.1.3. Histomorphometric evaluations of nerve regeneration through the three tubes

**Fig 5:**
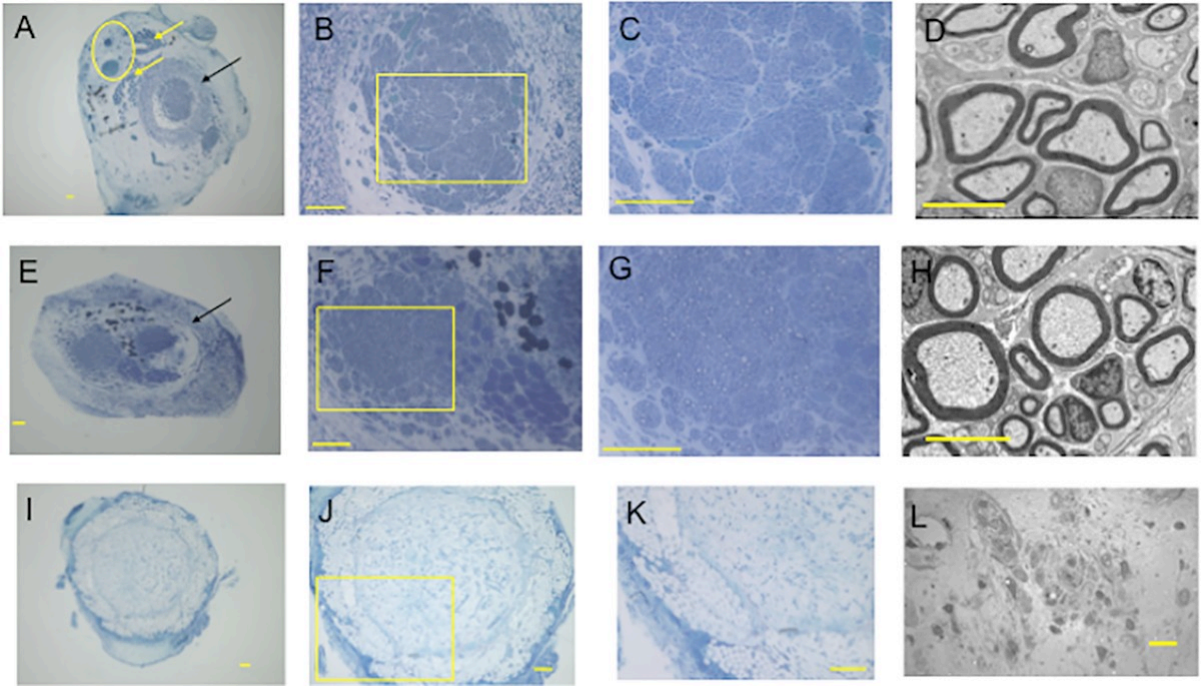
Histomorphometric study of E-tube, I-tube and N-tube groups. Toluidine blue staining of transverse semi-thin sections (1 μm thick) was used to perform optical microscopic examination **(A-C, E-G and I-K)** and transverse ultra-thin sections were used to perform transmission electron microscopic examination (**D**, **H and L**) of the regenerating nerves taken from the midpoint of each tube at 12 weeks. **A-D**: E-tube group. **E-H**: I-tube group. **I-L**: N-tube group. Yellow arrows in **A**: Muscular tissue included in the vascular pedicle. The yellow circle in **A** indicates the sural vascular pedicle attached to the outer surface of the tube. Black arrows in **A** and **E** indicate remnants of the PGA tube walls. Many regenerated axons were separated from the surround tissue by the tubular wall in E and I groups. No axons were found in N-tube group. Foreign body phagocytosis was found in some cells in **L**. The square shaped areas in **B, F** and **J** were magnified in **C, G** and **K**, respectively. **A-K except D and H**; bar = 100 μm. **D and H**; bar = 5 μm. L; bar = 10μm.

At 12 weeks, many regenerated nerve fibers were observed in both E-tube (Fig 5A–5D) and I-tube groups (Fig 5E–5H). In E-tube group, the regenerated nerve fibers were separated from the surrounding connective tissue by the PGA filament woven tubular wall. No remarkable scar tissue invasion from the extratubular space to the tubular lumen was found. (Fig 5A and 5B). Although hypocellular fluid tissue including macrophages with phagocytosis of foreign bodies was filled in the PGA tubular lumen, no axons were observed in N-tube group (Fig 5I–5L). Statistical comparison was thus performed between E-tube and I-tube groups. No significant difference was found in the mean myelinated axon number, mean myelinated axon diameter or mean myelinated thickness between I-tube and E-tube groups (Table 1).

### 4.2. Comparison of nerve regeneration through extratubularly vascularized PGA tubes containing DABLs seeded with BMSCs (TubeC+ group), extratubularly vascularized PGA tubes containing DABLs without BMSC transplantation (TubeC- group) and fresh autologous nerve segments (Auto group) in rat sciatic nerve with a 20mm deficit

#### 4.2.1. Extracellular matrix preservation in DABLs

**Fig 6:**
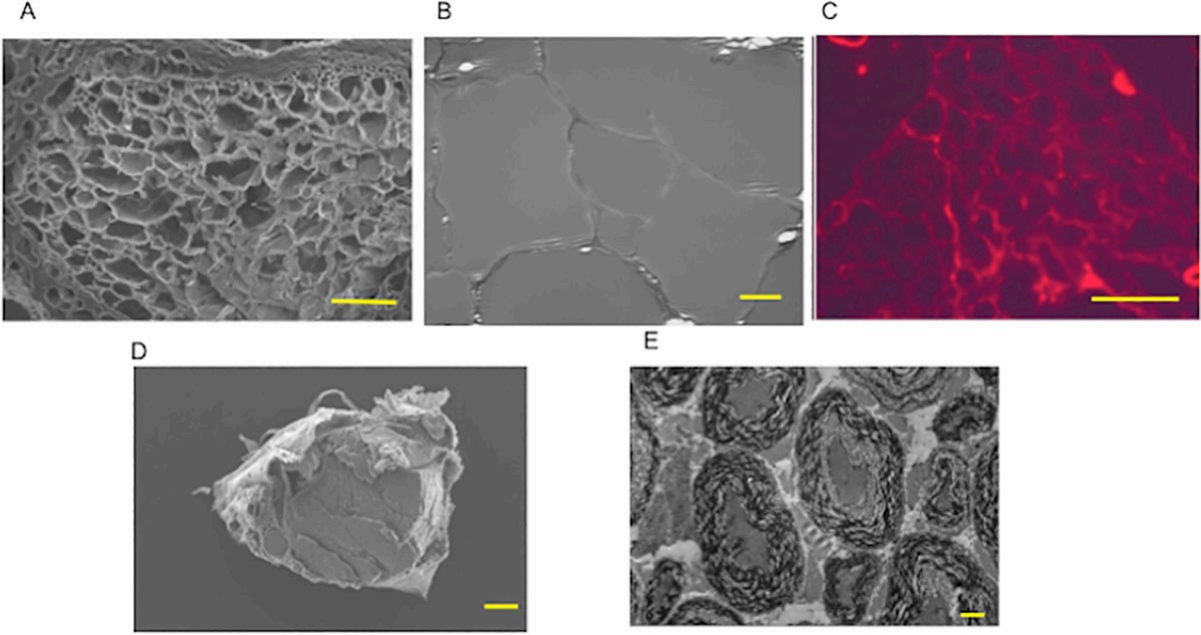
Preservation of extracellular matrix and electromicropic observation of chemically and thermally created DABLs. **A**: Scanning electron microscopic examination of the chemically created DABLs, which was used in the current study. Transverse section of the middle portion of the DABLs demonstrated honey-comb structure of basal lamellae. Bar = 100 μm. **B**: Transmission electron microscopic examination of the chemically created DABLs. Bar = 10 μm. **C:** Immunohistochemical examination for laminin on the transverse section of the chemically created DABLs. Laminin remained to some level in the DABLs. Bar = 100 μm. Scanning (**D**) and transmission (**E**) electron microscopic examinations of the DABLs created using the thermal (freeze and thaw) technique. The space between the basal lamellae was filled with cellular debris. The transmission microscopic examination revealed that the thermally created DABLs contained degenerated myelin structures and cell debris. Bar = 100 μm in **D** and 20 μm in **E**.

Electron microscopic examination revealed that there was almost no cellular debris in the chemically created DABLs (Fig 6A and 6B). In contrast, the space between basal lamellae was filled with cellular debris and the degenerated myelin structures were seen in the thermally created DABLs (Fig 6D and 6E). Immunohistochemical staining for laminin showed the laminin-positive structures along the basal lamellae, which indicated that some extracellular matrix containing laminin remained in the chemically created DABLs (Fig 6C). The amount of genomic DNA extracted from the chemically created DABLs was significantly smaller than that from the thermally created ones: 1.06 ± 0.25 μg/mg in the dry tissue of the chemically created DABLs and 1.63 ± 0.51 μg/mg in the dry tissue of the thermally created DABLs (p = 0.008).

#### 4.2.2. Immunogenicity of DABLs in TubeC+ and TubeC- group; comparison with Auto and Allo groups

**Fig 7:**
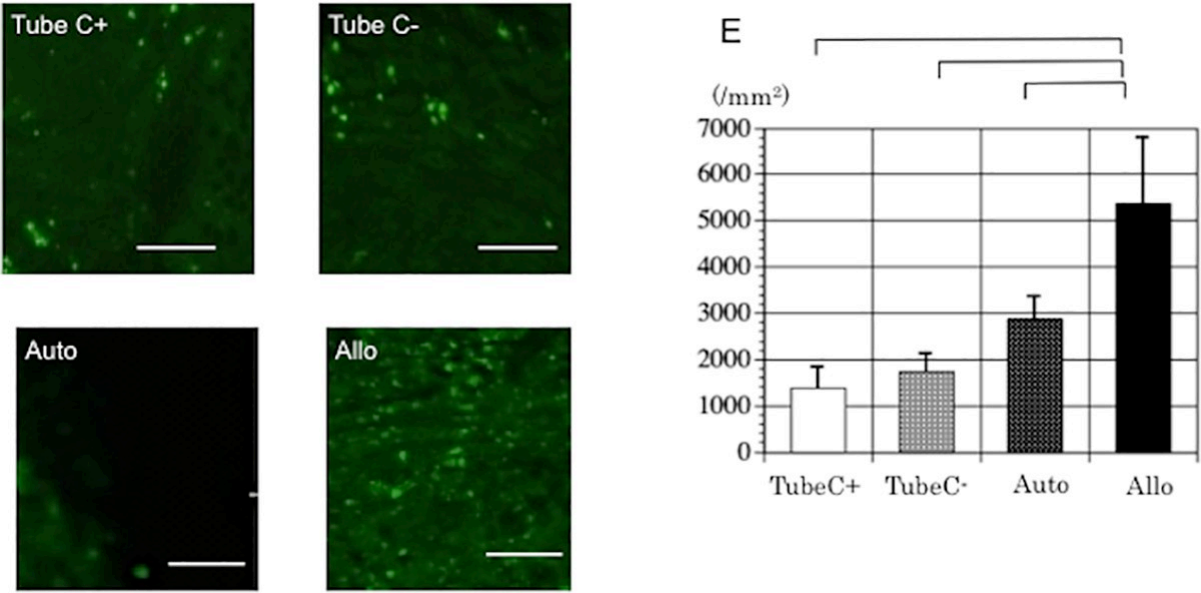
Assessment of immunological rejection of TubeC+, TubeC-, Auto and Allo groups. **Left;** Assessment of immunological rejection of nerve segments of Auto and Allo groups and DABLs of TubeC+, TubeC- groups. The transverse sections from the midportion of the conduits or grafts underwent immunohistochemistry for CD8a at 4 weeks. Bar = 100μm. **Right;** A graph demonstrating the mean numbers and standard deviations of the CD8 positive cells in TubeC+, TubeC-, Auto and Allo groups at 4 weeks after transplantation. The number of the CD8-positive cells was significantly greater in the Allo group than in the TubeC+, TubeC- and Auto groups. There was no significant difference among TubeC+, TubeC- and Auto groups. Brackets indicate significant difference.

According to the immunohistochemical studies, the mean numbers ± standard deviations of CD8a-positive cells in TubeC+, TubeC- Auto, and Allo groups were 1395±436/mm^2^, 1744±392/mm^2^, 2878±480/mm^2^ and 5363±1482/mm^2^, respectively at 4 weeks (Fig 7). There was significant difference between Allo group and each of the remaining three groups (p<0.001 each) (using one-way ANOVA with Bonferroni analysis). No significant difference was found among TubeC+, TubeC- and Auto groups. Considering these results, immunological rejection was not found against the DABLs transplanted in TubeC+ or TubeC- groups.

#### 4.2.3. Differentiation of BMSCs implanted with DABLs in the tube

**Fig 8:**
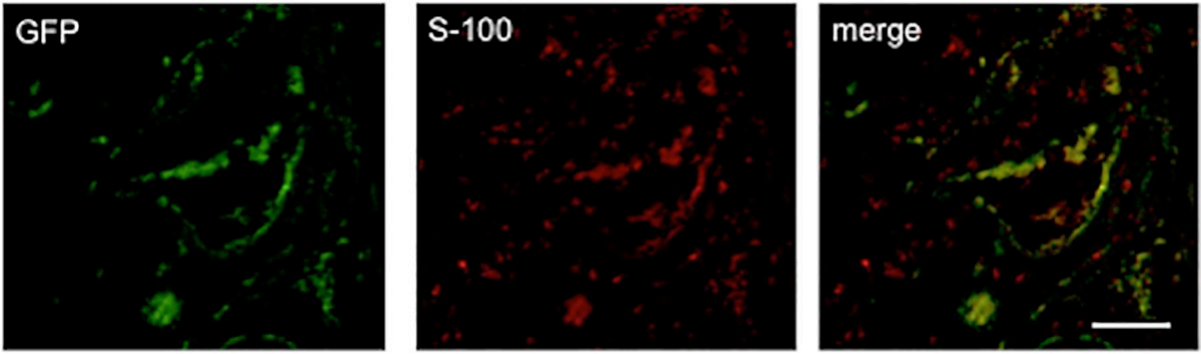
Differentiation of BMSCs in tubes of TubeC+ group 6 weeks after implantation. Differentiation of BMSCs in tubes of TubeC+ group 6 weeks after implantation of the BMSCs harvested from GFP-rats. **A**: Immunological staining for S-100 performed on a transverse section obtained from the midportion of the transplanted tube. **B**: Fluorescence microscopic examination for GFP positive cells. **C**: merged image of A and B. Some of GFP positive cells were immunopositive for S-100. Bar = 100μm.

In TubeC+ group with GFP-positive cell transplantation, GFP-positive cells were observed in the transplanted DABLs, indicating that transplanted BMSCs stayed within the tubes and survived for 6 weeks. Some of the GFP positive cells were also immunopositive with S-100, indicating that some transplanted BMSCs were induced to differentiate into Schwann cell-like cells in the DABLs in tubes in TubeC+ group (Fig 8).

#### 4.2.4. Comparison of nerve regeneration among TubeC+, TubeC- and Auto groups

**Fig 9:**
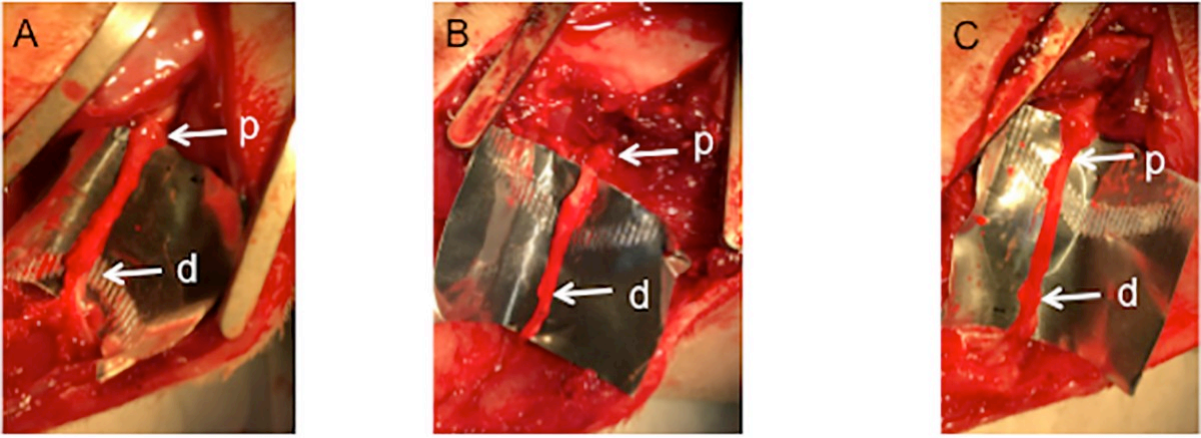
Macroscopic appearance of the transplanted tubes and nerve segments. Macroscopic appearance of the operated right sciatic nerves in TubeC+ (**A**), TubeC- (**B**) and Auto (**C**) groups at 24 weeks after surgery. **p**: proximal suture site, **d**: distal sutute site.

*4*.*2*.*4*.*1*. *Electrophysiological evaluations comparing among TubeC+*, *TubeC- and Auto groups*. In TubeC+ and TubeC- groups, the sciatic nerve deficits were successfully bridged by the PGF fiber tubes in all rats. (Fig 9).

At 12 weeks, the CMAPs were recorded in the pedal adductor muscles in all rats in all groups. The MNCV of Auto group was significantly greater than those of TubeC+ and TubeC- groups, while no significant difference was found between the two tube groups. No significant difference was found in the mean CMAP values among the groups (Table 2).

**Table 2:** Electrophysiological studies of TubeC+, TubeC- and Auto groups at 12 and 24 weeks.

*12W*	*TubeC+*	*TubeC-*	*Auto*
*MNCV*	^a^ 0.34±0.09	^b^0.29±0.08	^a^^,^^b^ 0.48±0.08
*CMAP*	0.17±0.09	0.12±0.02	0.19±0.09
*24W*	*TubeC+*	*TubeC-*	*Auto*
*MNCV*	0.58±0.15	0.56±0.15	0.63±0.09
*CMAP*	0.58±0.15	^c^ 0.38±0.16	^c^ 0.69±0.17

Note: Each value expresses the mean value ± standard deviation. The MNCVs and CMAP amplitudes were expressed as the ratios of those of the contralateral healthy limbs.

a; p = 0.014

b; p = 0.001

c; p = 0.003.

At 24 weeks, the mean CMAP amplitude was significantly greater in Auto group than TubeC- groups, although there was no significant difference between Auto and TubeC+ groups. No significance was found in the mean MNCVs among the three groups (Table 2).

**Fig 10:**
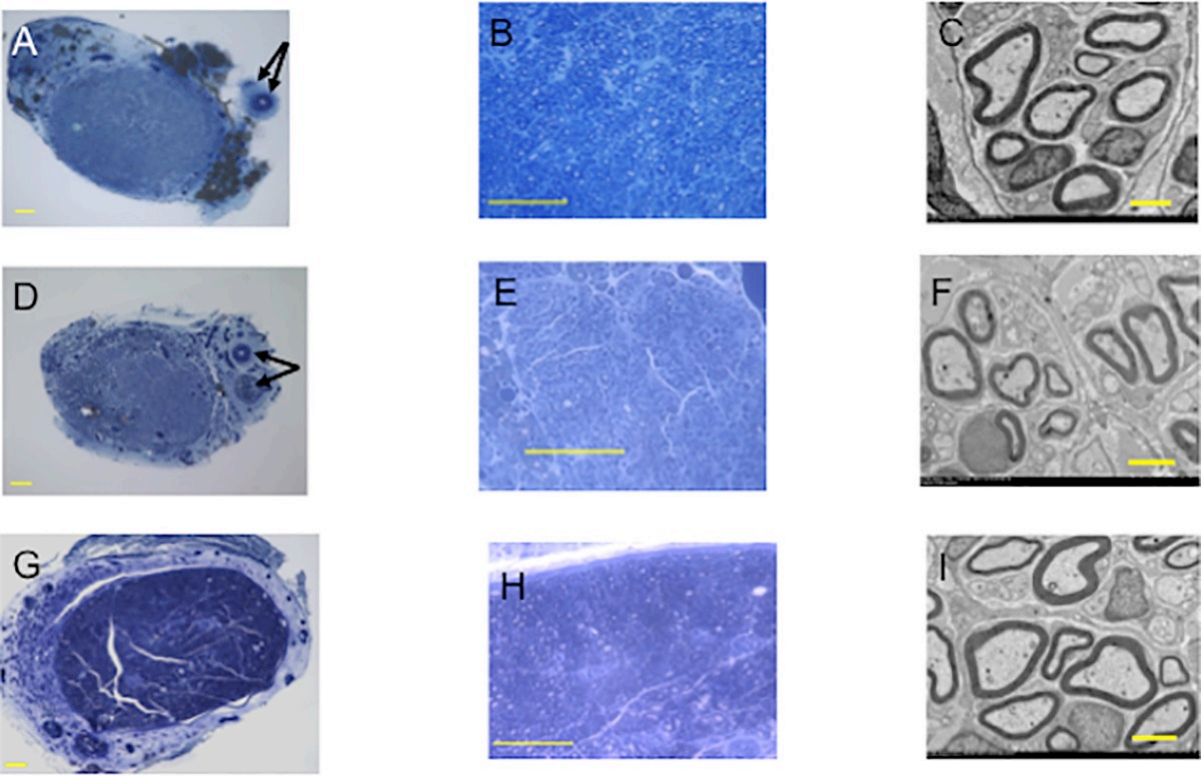
Morphometric study of Tube C+, Tube C- and Auto groups at 24 weeks. The semi-thin sections (**A, B**, **D**, **E**, **G**, **H**, bar = 100 μm) with toluidine blue staining and the ultra-thin sections (**C**, **F**, **I**, bar = 5 μm) of the regenerated nerves in the distal portion of the conduits or nerve segments at 24 weeks were shown. **A-C**: TubeC+ group. **D-F**: TubeC- group. **G-I**: Auto group. Black arrows indicated the patent arteries and veins of the sural vascular pedicles in **A** and **D**.

*4*.*2*.*4*.*2*. *Histological and morphometric evaluations*. In TubeC+ and TubeC- groups, the patency of the vascular bundles attached to the outside of the tubes was observed (Fig 10).

At 12 weeks, although no significant difference was found between TubeC+ and TubeC- groups, Auto group demonstrated significantly greater in the mean myelinated axon number and mean myelinated axon diameter than TubeC+ and TubeC- groups. In the mean myelin thickness, significant difference was found between Auto and TubeC- groups, but not between Auto and TubeC+ groups (Table 3).

**Table 3:** Histomorphometric studies of TubeC+, TubeC- and Auto groups at 12 and 24 weeks.

*12W*	*TubeC+*	*TubeC-*	*Auto*
*Axon Number*	^a^ 2199±512	^b^ 2048±648	^a^^,^^b^ 3794±481
*Axon Diameter (μm)*	^c^ 2.37±0.55	^d^ 2.28±0.57	^c^^,^^d^ 3.23±0.42
*Myelin Thickness (μm)*	0.46±0.22	^e^ 0.40±0.21	^e^ 0.66±0.15
*24W*	*TubeC+*	*TubeC-*	*Auto*
*Axon Number*	^f^^,^^g^ 4662±711	^g^^,^^h^ 3471±439	^f^^,^^h^ 5974±1205
*Axon Diameter (μm)*	^i^ 2.85±0.41	^j^ 2.37±0.26	^i^^,^^j^ 3.86±0.61
*Myelin Thickness (μm)*	^k^ 0.79±0.15	^l^ 0.64±0.06	^k^^,^^l^ 1.15±0.12

Note: Each value expresses the mean value ± standard deviation.

a; p<0.001

b; p<0.001

c; p = 0.009

d; p = 0.004

e; p = 0.043

f; p = 0.016

g; p = 0.031

h; p<0.001

i; p = 0.001

j; p<0.001

k; p<0.001

l; p<0.001.

At 24 weeks, Auto group was significantly superior to TubeC+ and TubeC- groups in all of the mean myelinated axon number, mean myelinated axon diameter and mean myelin thickness. In the mean myelinated axon number, TubeC+ group was significantly superior to TubeC- group (Table 3 and Fig 10)

*4*.*2*.*4*.*3*. *Reinnervated muscle weight*. At 24 weeks, the wet weight of the tibialis anterior muscles of the operated limbs (expressed as a ratio of the contralateral healthy limb) was 0.76±0.11, 0.69±0.06 and 0.59±0.11 in Auto, TubeC+ and TubeC- groups, respectively. Although the wet muscle weight in Auto group was significantly heavier than that in TubeC- group (p = 0.006), no significant difference was found between Auto and TubeC+ groups (p = 0.591) nor between TubeC+ and TubeC- groups (p = 0.115).

## 5. Discussion

In this study, capillaries passed through of the wall of the woven PGA fiber tube wall and extended into the regenerated nerve tissue formed within the tube at 4 weeks. Because no nerve regeneration was found within the tube without vascularity (N-tube group), the vascularity supplied by nerve stumps attached to the either end of the tube is not enough to regenerate axons through a water-permeable nerve conduit even though the interstump gap was only 5mm in rat sciatic nerves. Because no significant difference was found in capillary infiltration in the intrachamber neural tissue between I-tube and E-tube groups, the vascular bundle was attached on the outer surface of the tubes in TubeC+ and TubeC- groups. Successful nerve regeneration was obtained in rat sciatic nerves with a 20mm interstump gap through the extratubularly vascularized PGA tubes containing DABLs seeded with BMSCs (TubeC+ group) and those without BMSCs (TubeC- group). Although no significant difference was found between TubeC+ and Auto groups in the electrophysiological study or the wet muscle weight of the tibialis anterior muscles at 24 weeks, Auto group was significantly superior than TubeC+ and TubeC- groups in all of the parameters of the histomorphometric study. TubeC+ group demonstrated better nerve regeneration than TubeC- group at 24 weeks in the histomorphometric study (Tables 2 and 3), indicating that BMSC transplantation improved nerve regeneration within the tubes.

### 5.1. The role of the tube

From a clinical point of view, a silicone tube is structurally so solid that it cannot be used in the place near articular joints covered by thin subcutaneous tissue like hands and fingers, where peripheral nerve injury often occurs. The PGA conduit used in the current study was flexible, biodegradable and capillary-permeable. In the initial phase of nerve regeneration through a bioinert hollow conduit like a silicone tube, fibrin matrix is formed between the nerve stumps that are joined to either end of the conduit. Next, capillaries extend in the fibrin matrix from both nerve stumps, followed by Schwann cell migration and axon extension [23]. There is a limit of length of axon extension, which is about 10mm through a silicone tube in rat sciatic nerves [24]. Capillary extension within a nerve conduit might be one of the factors determining axon extension distance within the conduit [10, 25]. To increase the axon regeneration distance through a nerve conduit, a blood vascular bundle was inserted through the tubular lumen in several previous studies [2, 13, 25] and a capillary-permeable conduit accompanied by a vascular bundle was used in the present study.

The tube wall of a nerve conduit has another important role; to prevent scar tissue invasion into the tubular lumen [26]. The scar tissue invasion into the tubular lumen impedes the formation of the fibrin matrix within the tubular lumen. In the present study, regenerated nerve fibers were separated from the extratubular space by the PGA meshed tube wall. There was no remarkable scar invasion (Fig 5) but there was capillary infiltration into the tubular lumen of the PGA tubes (Fig 4). The woven structure of the PGA fibers prevented scar tissue invasion and tolerated capillary infiltration into the tubular lumen. There are few reports describing the relationship between nerve regeneration through a conduit and the size of molecules that can pass through the conduit wall. Previous authors reported that nerve regeneration through a conduit passing molecules less than 100 KDa was superior to that passing those less than 1000 KDa [27]. The PGA fiber tube used in this study pass molecules less than 600 KDa. It is still unknown about the optimal porous size created in the nerve conduit wall, which tolerates endotherial cell infiltration and prevents scar tissue invasion. The PGA conduit also exhibited the ability to retain some transplanted BMSCs inside the tubular lumen (Fig 8).

### 5.2. The role of DABLs

The histological examination revealed that the thermally created DABLs [2–5] contained degenerated myelin structures and cellular debris (Fig 6D and 6E), while the DABLs created using the chemical surfactants [6] left only basal lamellae with almost no cell debris (Fig 6A and 6B), which could reduce the risk of pathologic organism transmission through the DABL transplantation. The genomic DNA study demonstrated that the DNA amount left in the chemically created DABLs was significantly smaller than that in the thermally created DABLs (about 65% of the latter). The transplantation of chemically created DABLs can reduce the risk of organism transmission, compared with the transplantation of thermally created DABLs. However, it is still impossible to eliminate organism transmission completely even in the transplantation of chemically created DABLs.

The wall of the PGA fiber tube has the permeability of water as well as capillaries. In a water-permeable hollow nerve conduit, the fibrin matrix formation is impeded because of the leakage of the matrix through the tubular wall. Nerve regeneration did not happen even in a 5mm-interstump gap through a water-permeable tube in rat sciatic nerves, when the source of capillary supply was limited to the nerve stumps joined to either end of the tube (N-tube group). Therefore, in the process of nerve regeneration through a water-permeable nerve conduit, some structures that keep the fibrin matrix containing some neurochemical factors inside the tube are necessary. In the current study, the fibrin matrix may have been retained in the space between the lamellae of the DABLs due to the surface tension of the fibrin matrix. In addition, the DABLs also acted as a scaffold for axon extension, Schwann cell migration [3] and the retention of the transplanted BMSCs in the tubular lumen.

In the present study, the DABLs made from DA rat sciatic nerve segments were transplanted to Lewis rats that had major histocompatibility mismatch with DA rats. However, after the decellularizing process, because the DABLs lost prominent immunological antigenicity, the number of cellular hazardous T cells was significantly smaller in the DABLs in TubeC+ group than that in the fresh allogenic nerve segments (Allo group) and almost equal to that in the autologous fresh nerve segments (Auto group) (Fig 7). Because laminin (a primary component of the extracellular matrix) preserved in the DABLs is a factor to promote nerve regeneration (Fig 6C) [28, 29], the reduced antigenicity and laminin preservation of the DABLs might have led to successful nerve regeneration through the PGA tubes with the DABL transplantation.

### 5.3. The role of transplanted BMSCs

BMSCs transplanted inside a nerve conduit are reported to have several functions to facilitate nerve regeneration within the conduit including homing ability [30–32], production of neurotrophic factors [14, 33–35], and differentiation into Schwann cell-like cells [2, 13, 18–20, 29, 36, 37] (Fig 8). Some authors reported that the transplanted BMSCs functioned as a mediator of immune reaction, which can prevent allogeneic reaction of the transplanted DABLs [38, 39].

It can be anticipated that some transplanted cells might have leaked out of the PGA fiber tube. Considering better nerve regeneration of TubeC+ group compared to that of TubeC- group, some transplanted BMSCs remained in the tubular lumen and differentiated into Schwan cell-like cells that might have promoted nerve regeneration within the tubes (Fig 8).

Several limitations are associated with the BMSCs used in this study. BMSCs with 5–7 passages were used in the current study as reported in the previous study [2]. Human MSCs subjected to extensive in-vitro passage can undergo morphological, phenotypic, and genetic changes [40, 41]. It is reported that MSCs begins to enlarge after passage 5 in the culture [41]. In our previous [2] and present studies, we have not observed a remarkable decrease of the proliferative rate of BMSCs or increase of the cell size irregularity during the BMSC culture, because we might have harvested BMSCs from 7-week young rats. Considering the risks of karyotypic anomalies of BMSCs during the cell culture, we should have used BMSCs with 4 or fewer passages or checked the CD antigen surface markers of the BMSCs before the transplantation as Madsen et al. recommended [42, 43]. In the TubeC+ group, when BMSCs were injected into the DABL, a small amount of FBS that has the potential to accelerate nerve regeneration was also injected. We also consider a possibility that FDS might have promoted nerve regeneration when we interpret the results of TubeC+ group.

In this study, we demonstrated some transplanted BMSCs differentiated into Schwann-cell-like cells, although the differentiated BMSCs were not quantified. In future, we must investigate the percentage of the transplanted BMSCs differentiate into Schwann cell-like cells in the nerve conduits and whether the Schwann cell-like cells really function like normal Schwann cells.

### 5.4. Nerve regeneration through our biodegradable tube in comparison with autogenous nerve graft

At 24 weeks, there was no significant difference in electrophysiological study between TubeC+ and Auto groups, although all histomorphometric parameters of Auto group were significantly superior to those of TubeC+ group. Mean values of the 24 week-histomorphometric parameters in TubeC+ group ranged almost 70–85% of those of Auto group. However, in the current study, the nerve grafting was performed in extremely good conditions that can hardly be experienced in actual clinical settings. Size match between the graft and host nerve stumps was almost perfect. The nerve gap was bridged by a single cable graft. There was no time delay between the nerve transection and repair.

Nerve regeneration capability of TubeC+ tubes is extremely superior to that of the current commercialized artificial nerves that are indicated for repair of a nerve deficit less than 3cm in humans [1], because good motor recovery was found in TubeC+ group in a 2 cm-gap in rodent sciatic nerve, which almost corresponds to a 6 cm-gap in primate or human peripheral nerves [44]. Considering the above mentioned situations, the extratubularly vascularized PGA fiber conduit containing DABLs and BMSCs may be an alternative to a fresh autologous nerve graft.

There have been several studies of peripheral nerve regeneration using decellularized nerve grafts combined with stem cell transplantation. Li et al. transplanted a decellularized allogenic nerve graft seeded with BMSCs to bridge a 30mm-long sciatic nerve defect in rabbits and obtained nerve regeneration similar to that in autogenous nerve grafts [45]. Nakada et al. wrapped a decellularized nerve with a sheet seeded with adipose derived stem cells and transplanted it to bridge a 15mm-rat sciatic nerve defect and reported that nerve regeneration in their materials was compatible with that in autogenous nerve grafts [46]. Because of the difference of animal species and methods evaluating nerve regeneration, it was unknown which nerve induction material was the most promotive for peripheral nerve regeneration. With regard to nerve conduits, clinical availability is also important. In our previous study [2], a silicone tube containing a blood-vessel pedicle and a decellualarized allogenic nerve graft seeded with BMSCs was transplanted to bridge a 20mm-rat sciatic nerve gap (VDB group). Although there was no significant difference in the 24 week-electrophysiological and histomorphometric outcomes between VDB group and TubeC+ group (http://id.nii.ac.jp/1391/00021905/), the PGA fiber tubes are more applicable to clinical settings than silicone tubes because of the biodegradability and structural flexibility of the PGA tubes.

### 5.5. Possibility of clinical application of TubeC+ tubes and future study

Several technical modifications are necessary to apply TubeC+ tubes clinically. First, BMSC culture needs some special facilities that are designed specifically to perform ex-vivo cell culture safely. Cell culture also needs time for cell passages. It is known that repeating cell passages increases the risk of tumor genesis of the stem cells [39, 40]. Recently, autologous BMSCs can be taken from the iliac bone marrow liquid in situ using a special cell filter apparatus without using cell culture technique, although some modification will be needed to obtain enough BMSCs to promote nerve regeneration [47, 48]. Second, vascularity of the recipient site is important when a TubeC+ tube is used as a nerve conduit. If vascularity is poor at the recipient site, some vascular pedicle is harvested and transplanted along the tube as we did in this study or vascularized tissue such as fascia, muscle and adipose tissue is transplanted around the tube. With the use of these techniques, transplantation of the PGA fiber conduits containing DABLs and BMSCs can be performed in usual operation theaters not equipped with any special instruments or facilities for cell culture. In future studies, we will transplant TubeC+ tubes to a long nerve deficit in larger animals including canines.

In conclusion, the PGA fiber tubes have good flexibility, biodegradability and capabilities to allow capillary infiltration and prevent scar tissue invasion. Because the PGA tube containing DABLs seeded with BMSCs demonstrated good nerve regeneration that was compatible with fresh autologous nerve grafts electrophysiologically, they are expected to be one of the promising options for repair of peripheral nerve deficits.

## Supporting information

S1 File Minimal data set. (DOCX)
