# Supplementary material for: Bone marrow-derived mesenchymal stem cells transplanted into a vascularized biodegradable tube containing decellularized allogenic nerve basal laminae promoted peripheral nerve regeneration; can it be an alternative of autologous nerve graft?
Source: PLoS One. 2021 Aug 31;16(8):e0254968. doi: 10.1371/journal.pone.0254968 (PMC8407554; doi:10.1371/journal.pone.0254968)
Supplement: S1 File — (DOCX) [file pone.0254968.s001.docx]

S1 File. MINIMAL DATA SET

We added a minimal data set regarding our description of line 6-9 in page 53.

Histomorphometric study

| *24W* | *TubeC+* | *VBD* | *p* |
| --- | --- | --- | --- |
| *Number* | *8* | *8* | *-* |
| *MNCV* | 0.58±0.15 | 0.64±0.12 | n.s. |
| *CMAP* | 0.58±0.15 | 0.60±0.18 | n.s. |

Electrophysiological study

| *24W* | *TubeC+* | *VBD* | *p* |
| --- | --- | --- | --- |
| *Number* | *8* | *8* | *-* |
| *Axon Number* | 4662±711 | 4850±908 | n.s. |
| *Axon Diameter (μm)* | 2.85±0.41 | 2.90±0.36 | n.s. |
| *Myelin Thickness (μm)* | 0.79±0.15 | 0.77±0.09 | n.s. |

n.s.: p.>0.05

In the present study, the histological and electrophysiolocical comparison was performed between TubeC+ and VBD groups using chemically created DABLs. In the previous study reported by Kaizawa et al. in 2017 [2], thermally created DABL was used in VBD group.
